# Particles in the Eye

**Published:** 1882-04

**Authors:** 


### PARTICLES IN THE EYE.

OLD and young persons are often sorely troubled by small, hard particles of matter that get under the eyelids. When children suffer in this way their parents may not even suspect the cause of the trouble. The irritation may go on increasing for years, for the inflammation strongly resembles catarrhal conjunctivitis, which has quite a different cause. The conjunctiva (as the termination itis in medicine always means "inflammation of," conjunctivitis means inflammation of the conjunctiva) is a mucous membrane which begins near the edge of the lids, upper and lower, lines them, and then, turning back, covers also the eyeball. It thus forms two sacs. It is exceedingly sensitive, and is very liable to inflammation of various kinds, all painful, and some very difficult of cure. A foreign body beneath the eyelid soon inflames it. Such a body beneath the upper lid is not as readily detected as one beneath the lower, and it is harder to remove it. A child that had long suffered from what was supposed to be catarrhal inflammation, and for which it had been energetically treated, only to grow worse, was brought to Dr. Broosa, Professor of Ophthalmology in the New York University. On turning back the child's upper eyelid, the source of the trouble was found in a small bud of a cherry tree. Relief and cure followed its removal. In all such cases the main thing to do is to evert the lid. The lower lid is easily turned over the finger. If the particle is beneath the upper lid, press the lid against the eyebrow and have the patient look down. Then seize the eyelashes and edge of the lid and turn the lid quickly over the thumb. Remove the speck with a handkerchief and show it to the patient; for he will often feel for some time as if the object were still in the eye.

